# Occurrence of mycobacteria in bovine milk samples from both individual and collective bulk tanks at farms and informal markets in the southeast region of Sao Paulo, Brazil

**DOI:** 10.1186/1746-6148-9-85

**Published:** 2013-04-24

**Authors:** Marília Masello Junqueira Franco, Antonio Carlos Paes, Márcio Garcia Ribeiro, José Carlos de Figueiredo Pantoja, Adolfo Carlos Barreto Santos, Marcelo Miyata, Clarice Queico Fujimura Leite, Rodrigo Garcia Motta, Fernando José Paganini Listoni

**Affiliations:** 1 Department of Veterinary Hygiene and Public Health, School of Veterinary Medicine and Animal Science, UNESP – Univ Estadual Paulista, Box 56018618-970, Botucatu, State of Sao Paulo, Brazil; 2 Laboratory of Micobacteriology, School of Pharmacy Sciences, UNESP – Univ Estadual Paulista, 14800-901, Araraquara, State of Sao Paulo, Brazil

**Keywords:** *Mycobacterium* spp, Bovine, Raw milk, Bulk tanks, Public health, Brazil

## Abstract

**Background:**

*Mycobacterium* spp. is one of the most important species of zoonotic pathogens that can be transmitted from cattle to humans. The presence of these opportunistic, pathogenic bacteria in bovine milk has emerged as a public-health concern, especially among individuals who consume raw milk and related dairy products. To address this concern, the Brazilian control and eradication program focusing on bovine tuberculosis, was established in 2001. However, bovine tuberculosis continues to afflict approximately 1,3 percent of the cattle in Brazil. In the present study, 300 samples of milk from bovine herds, obtained from both individual and collective bulk tanks and informal points of sale, were cultured on Löwenstein-Jensen and Stonebrink media. Polymerase chain reaction (PCR)-based tests and restriction-enzyme pattern analysis were then performed on the colonies exhibiting phenotypes suggestive of *Mycobacterium* spp., which were characterized as acid-fast bacilli.

**Results:**

Of the 300 bovine milk samples that were processed, 24 were positively identified as *Mycobacterium* spp.

Molecular identification detected 15 unique mycobacterial species: *Mycobacterium bovis*, *M. gordonae*, *M. fortuitum, M. intracellulare, M. flavescens, M. duvalii, M. haemophilum, M. immunogenum, M. lentiflavum, M. mucogenicum, M. novocastrense, M. parafortuitum, M. smegmatis, M. terrae* and *M. vaccae*. The isolation of bacteria from the various locations occurred in the following proportions: 9 percent of the individual bulk-tank samples, 7 percent of the collective bulk-tank samples and 8 percent of the informal-trade samples. No statistically significant difference was observed between the presence of *Mycobacterium* spp. in the three types of samples collected, the milk production profiles, the presence of veterinary assistance and the reported concerns about bovine tuberculosis prevention in the herds.

**Conclusion:**

The microbiological cultures associated with PCR-based identification tests are possible tools for the investigation of the presence of *Mycobacterium* spp. in milk samples. Using these methods, we found that the Brazilian population may be regularly exposed to mycobacteria by consuming raw bovine milk and related dairy products. These evidences reinforces the need to optimize quality programs of dairy products, to intensify the sanitary inspection of these products and the necessity of further studies on the presence of *Mycobacterium* spp. in milk and milk-based products.

## Background

Milk is an important source of proteins, sugars, lipids and other nutrients for humans. However, these nutrients can also serve as substrates for pathogenic microorganisms. The traditional consumption of homemade dairy products, and especially cheeses, that are composed of non-heat-treated milk poses a serious risk to public health [1]. It is estimated that more than 90 percent of all of the cases of illness related to the consumption of dairy products are of bacterial etiology, and at least 21 different diseases of this type have been identified [2].

Over the last three decades, approximately 75 percent of emerging infectious diseases in humans have been zoonoses. The increasing interdependence of humans on animals and animal products has been determined to be the most critical risk factor for such infectious diseases [3]. Mycobacterial infections are among the most debilitating conditions affecting humans and animals because these infections are both chronic and progressive [4]. Among such infections, tuberculosis is one of the main zoonotic diseases transmitted by bovine milk [5]. Microorganisms of the *Mycobacterium* genus, such as *Mycobacterium bovis*, are highly able to survive in bovine milk and other dairy products. These microbes can be found in the form of viable bacilli in cream cheese and yogurt produced from raw milk for over 14 days and in butter for over 100 days [6]. Recently, the presence of these opportunistic, pathogenic bacteria in milk has emerged as a public-health concern, especially among individuals who consume raw milk and related dairy products [2].

Considering these health concerns and international trade requirements, many countries have implemented national programs for the control and eradication of bovine tuberculosis [7]. In Brazil, the National Program for Control and Eradication of Brucellosis and Tuberculosis (‘Programa Nacional de Controle e Erradicação da Brucelose e Tuberculose’ - PNCEBT) was established in 2001, with the aim of reducing the incidence of tuberculosis in bovine and bubaline herds [8]. Brazilian legislative measures now require the regulation of the storage, collection and transport of refrigerated raw milk [9]. The country has also established a National Program of Milk Quality to increase the quality of milk production. Despite this program, however, small farmers are still selling unpasteurized milk that has not undergone sanitary inspection to individuals who prefer this type of dairy product. National surveys have confirmed the habit of consumption of milk without any heat treatment [10], and there are no validated laboratory methods that allow the certification of such untreated milk or dairy products as “free of viable mycobacteria” [6].

The aim of the current study was to investigate the occurrence of mycobacteria in bovine raw-milk samples, obtained from both individual and collective bulk tanks and informal trade in the southeast region of Sao Paulo, Brazil, using microbiological cultures and polymerase chain reaction (PCR)-based testing. The specific objectives of this study included the comparison of the presence of mycobacteria in various sample types, the use of bovine milk samples from herds to evaluate the risk of human contact with these bacteria and the use of PCR and restriction-enzyme pattern analysis (PRA) to identify the *Mycobacterium* spp.

## Methods

### Ethical statement

The study was approved by the Ethics Committee on the Use of Animals of the School of Veterinary Medicine and Animal Science of Sao Paulo State University (FMVZ-UNESP/Botucatu), under the protocol number 156/2010-CEUA.

### Enrolled farms

This study included small- and medium-sized farms in the southeast region of Sao Paulo state, a major milk-producing area of Brazil, inserted into the largest national consumer market. The farms, which were randomly chosen, used either automatic or manual milking systems and possessed between two and 200 lactating animals. The herds were composed of the Holstein, Jersey or Girolando breeds or of crossbreeds with the ability to produce milk and exhibited a median milk production of 12 liters per day.

Epidemiological data on the herds were obtained using a questionnaire administered to farmers and milk-sellers that requested information on the following: the city where the farm was located, the type of milking system, the daily total volume milked (in liters), the mean volume milked (in liters), the number of lactating animals, the breeds of the lactating animals, the presence or absence of veterinary assistance and whether there were concerns about tuberculosis prevention in the herd. In the case of collective bulk tanks, which are used by more than one farmer to deliver milk, the number of delivering producers per bulk tank was also ascertained.

### Sample collection

One hundred samples were collected directly from the individual bulk tanks, and 100 were collected from the collective bulk tanks. One hundred samples were also collected at informal points of sale in the same region. In this case, milk was bought directly from informal sellers. This sample collection included 25 different cities in the southeast region of Sao Paulo, Brazil.

A total volume of 100 milliliters was collected per sample, and each of the 300 samples was placed in a sterilized collection flask at the time of collection. The samples were immediately stored on ice in isothermal boxes (4 to 8 degrees Celsius) until their arrival at the laboratory.

### Mycobacterial cultures

To avoid contamination or biological risk, the samples were processed in a class II biological safety cabinet that had been cleaned with 10 percent sodium hypochlorite and 70 percent alcohol, followed by exposure to ultraviolet light for 30 minutes, with the air circulation system turned on. An 8-milliliter aliquot of each sample was then transferred into a screw-top centrifuge tube for high-speed centrifugation (10.000 rotations per minute for 20 minutes). During this process, the cells and fat from the samples were concentrated, and the remaining serum was removed. The Petroff decontamination method, using 4% sodium hydroxide and 4% sulfuric acid [11], was performed on the mixture of fat and protein sediment derived from each sample to be used for mycobacterial culture.

The cultures were maintained on Löwenstein-Jensen and Stonebrink media, incubated aerobically at 37 degrees Celsius for 90 days and examined weekly. Any colonies exhibiting phenotypes suggestive of mycobacteria were stained using the Ziehl-Neelsen method and maintained on the same media at room temperature and hidden from light until PRA was performed.

### PCR-based species identification

For the PCR-based species identification using PRA, DNA extraction was performed by thermolysis. A loopful (10 microliters) of mycobacteria was suspended in 300 microliters of Tris-EDTA buffer (pH 8.0) in DNase/RNase-free microcentrifuge tubes, boiled for 10 minutes and frozen (−20 degrees Celsius) for 10 minutes. These boiling and freezing processes were repeated three times. To separate the DNA, the microtubes were centrifuged (12.000 rotations per minute for 5 minutes). Three microliters of the supernatant containing the DNA, 25 picomoles of each primer and 34 microliters of PCR Master Mix (2X)™ (Fermentas™, Thermo Fisher Scientific, Vilnius, Lithuania) were then combined for PCR. This reaction amplified a 439-bp fragment within the *hsp*65 gene using the primers Tb11 (5′–ACCAACGATGGTGTGTCCAT–3′) and Tb12 (5′–CTTGTCGAACCGCATACCCT–3′) [12].

The PCR protocol consisted of a denaturation step at 95 degrees Celsius for 10 minutes, followed by 45 cycles at 94 degrees Celsius for 1 minute, 60 degrees Celsius for 1 minute and 72 degrees Celsius for 1 minute and a final extension step at 72 degrees Celsius for 10 minutes (PTC-100 Thermal Cycler, MJ Research™, Basel, Switzerland). The electrophoresis of 3 microliters of the PCR product was then performed on an agarose gel (1 percent) using a 100-bp molecular-weight marker (Invitrogen™, Carlsbad, United States of America) to confirm the amplification of the 439-bp fragment. Ten to 15 microliters of the amplified product was digested with the *BstE*II and *Hae*III restriction enzymes according to the manufacturer’s instructions (Fermentas™). The resulting restriction fragments were separated by electrophoresis on an agarose gel (4 percent) using 25- and 50-bp molecular-weight markers (Invitrogen™). The fragment sizes were estimated using Alpha Ease-Alpha Innotech software (version 6.0) [Alpha Innotech™, San Leandro, United States of America] and analyzed using internet Prasite [13].

The amplification of the *hsp*65 gene cannot differentiate between *M. tuberculosis* complex (MTBC) species due to these species’ close genetic relationship. Therefore, isolates belonging to the MTBC have been analyzed by amplifying a 1020-bp fragment of the *gyr*B gene using PRA [14]. In this case, 3 microliters of mycobacterial DNA was added to 44 microliters of PCR Master Mix (2X)™, and subjected to PCR using 10 picomoles of each of the primers MTUBf (5′–TCGGACGCGTATGCGATATC–3′) and MTUBr (5′–ACATACAGTTCGGACTTGCG–3′). The PCR protocol consisted of an initial denaturation step for 10 minutes at 95 degrees Celsius; 35 amplification cycles, each consisting of 1 minute at 94 degrees Celsius, 1 minute at 65 degrees Celsius and 1,5 minutes at 72 degrees Celsius; and a final extension step of 10 minutes at 72 degrees Celsius. Electrophoresis on an agarose gel (1 percent) confirmed the amplification of the fragment. The amplicon was further digested using the restriction enzymes *Rsa*I, *Taq*I and *Sac*II according to the manufacturer’s instructions (Fermentas™). The restriction digests were separated on an agarose gel (2 percent) using 50- and 100-bp DNA molecular-weight markers (Invitrogen™). The separated fragments of the *gyr*B gene were analyzed using Alpha Ease-Alpha Innotech software (version 6.0) [Alpha Innotech™] and compared with the patterns that were described by Chimara et al. [14].

### Data analysis

For the data analysis, continuous variables were categorized based on their median values. The total volume milked per day was classified as high (>330 liters) or low (<330 liters), and the mean volume milked per day was classified as high (>12 liters) or low (<12 liters). The number of lactating animals in each herd was categorized as many (>11 cows) or few (<11 cows). Similarly, the number of farmers delivering milk to each collective bulk tank was separated into two categories: many (>14 farmers) or few (<14 farmers).

Summary statistics were calculated to describe the characteristics of the farms enrolled in the study. Chi-squared or Fisher’s exact tests were used to identify the farm-related factors that were associated with the isolation of *Mycobacterium* spp. A logistic regression was then used to estimate the adjusted associations in a multivariable model, and backward variable selection was used to select a possible final model. The data were analyzed using R software (version 2.15.1) [15].

## Results and discussion

Information regarding the daily total volume milked (in liters), the mean volume milked (in liters), the number of lactating animals, the breeds of the lactating animals, the presence of veterinary assistance and whether concerns existed about the prevention of tuberculosis in the herd was obtained for 228 of the 300 samples that were collected. This information was not collected for all of the samples because the informal milk sellers were not always aware of each product’s origin. The number of farmers who delivered milk to each collective bulk tank was also considered. The sampled population was highly heterogeneous, the descriptive analyses of these data are summarized in Table 1.

**Table 1:** Population characteristics of 228 enrolled farms on southeast region of Sao Paulo state, Brazil

	**Mean**	**SD**	**Median**	**Min**	**Max**
Total volume milked/day (liters)	443.95	397.15	330	15	3000
Mean volume milked/day (liters)	25.31	25.05	12	2	125
Lactating animals	23.53	27.73	11	2	200
Farmers/collective bulk tank	14.52	7.62	14	2	33

*SD* = Standard deviation.

*Min* = Minimum value.

*Max* = Maximum value.

Of the 300 samples that were processed, 24 tested positive for *Mycobacterium* spp. Molecular methods identified 15 distinct species among the isolates, as shown in Table 2. One isolate belonged to the MTBC (*M. bovis* subsp. *bovis*), and the other isolates included the environmental nontuberculous mycobacteria (NTM) *M. gordonae*, *M. fortuitum, M. intracellulare, M. flavescens, M. duvalii, M. haemophilum, M. immunogenum, M. lentiflavum, M. mucogenicum, M. novocastrense, M. parafortuitum, M. smegmatis, M. terrae* and *M. vaccae*. The species that was most frequently isolated was *M. gordonae* type 9, and the second most frequent was *M. haemophilum* type 1.

**Table 2:** ***Mycobacterium *spp. isolated from 300 bovine raw milk samples on Sao Paulo state, Brazil**

	**Individual bulk tanks**	**Collective bulk tanks**	**Informal trade**
*M. bovis* subsp *bovis*	01/100	-	-
*M. duvalii* type 1	-	01/100	-
*M. flavescens* type 1	-	01/100	-
*M. fortuitum* type 1	01/100	-	-
*M. gordonae* type 1	01/100	-	-
*M. gordonae* type 2	01/100	-	-
*M. gordonae* type 9	03/100	-	01/100
*M. haemophilum* type 1	-	01/100	02/100
*M. immunogenum* type 1	-	-	02/100
*M. intracellulare* type 1	01/100	-	-
*M. lentiflavum* type 2	-	-	01/100
*M. mucogenicum* type 2	01/100	-	-
*M. novocastrense* type 1	-	02/100	-
*M. parafortuitum* type 1	-	01/100	-
*M. smegmatis* type 1	-	01/100	-
*M. terrae* type 3	-	-	01/100
*M. vaccae* type 1	-	-	01/100

Nine percent of the samples from the individual bulk tanks yielded *Mycobacterium* spp.-positive cultures, whereas 7 percent of the samples from the collective bulk tanks and 8 percent of the informal-trade samples resulted in positive cultures (Table 3). No statistically significant difference was observed between the number of samples that tested positive by mycobacterial culture and the epidemiological factors that were considered. These factors included the types of samples collected, the presence or absence of veterinary assistance on the farm, the concerns about tuberculosis prevention in the herd, the total volume milked per day, the mean volume milked per day, the number of lactating animals and the number of farmers delivering milk to each collective bulk tank, as shown in Table 3.

**Table 3:** **Univariate analysis of risk factors for the isolation of *Mycobacterium *spp**

	**Isolation of *Mycobacterium *spp**			
**Risk factors**	**n**	**%**	**Total**	*P*-value*
Milk sample origin				0,87
Individual bulk tank	9	9	100	
Collective bulk tank	7	7	100	
Informal trade	8	8	100	
Veterinary assistance				0,36
Individual bulk tank	55	55	100	
Collective bulk tank	35	35	100	
Informal trade	3	11	28	
Concerns on bovine tuberculosis prevention				0,24
Individual bulk tank	42	42	100	
Collective bulk tank	32	32	100	
Informal trade	4	14	28	
Total volume milked/day				0,18
High	113	49	228	
Low	115	50	228	
Mean volume milked/day				0,43
High	114	50	228	
Low	114	50	228	
Lactating animals				0,87
Many	116	51	228	
Few	112	49	228	
Farmers/collective bulk tank				0,86
Many	54	54	100	
Few	46	46	100	

*Pearson’s Chi-squared test, association with *Mycobacterium* spp. isolation.

The screening tests of milk samples has been used to limit the spread of infectious diseases [16,17], including certain diseases caused by mycobacteria, such as paratuberculosis [18]. As the use of bulk-tank milk samples for screening is less labor-intensive and more cost-effective than individual-tank sampling [19], this approach represents an important development in dairy research.

Previous studies have addressed the identification of mycobacteria in milk samples collected from both individual and collective bulk tanks. In one study, opportunistic mycobacteria were recovered from more than 55 percent of the samples collected from milking platforms [20]. In another study, in 1975, the presence of *M. marinum*, *M. scrofulaceum*, *M. gordonae*, *M. flavescens* and *M. fortuitum* was reported in 68.8 percent of 51 raw-milk samples [21]. Years later, researchers isolated mycobacteria from 4.3 percent of 209 milk samples collected from Indian farmers and traders [22]. In yet another study, saprophytic mycobacteria were identified in 13 percent of 285 milk samples [23]. Species from the MTBC have also been isolated from milk; in one case, 16 isolates of *M. bovis* and 8 isolates of *M. tuberculosis* were obtained from 543 milk samples collected from bulk tanks [24]. Additionally, a study conducted in Korea demonstrated that 4.5 percent of the collected milk samples tested positive for *M. bovis*. However, neither molecular characterization nor microbiological cultures of the samples were included by the researchers [19]. These data agree with the results obtained in the present study, in which both saprophytic and pathogenic mycobacteria were identified in bovine milk samples.

Species from the genus *Mycobacterium* are recognized as major pathogens transmitted between the environment, wildlife, livestock and humans. *M. bovis* represents a serious problem in the international trade of animals and animal products, resulting in great economic losses to livestock [25]. Zoonotic tuberculosis in man is attributed mainly to *M. bovis* and occasionally to *M. tuberculosis*, and this disease is mainly transmitted through milk. Therefore, certain habits, such as the consumption of raw bovine milk, may predispose individuals to these infections [26]. Although *M. bovis* does not multiply in milk or does so very slowly, the large number of mycobacteria that are secreted into the milk of one animal with tuberculous mastitis is generally sufficient render the homogenized milk from 100 lactating cows [6]. Given such risks and the fact that Brazil has the largest commercial cattle herd in the world, it is essential to ensure the high quality and low health risk of animal products destined for customers, who are increasingly exigent [8].

The ability of NTM to trigger disease is clearly documented in the literature, and concern about NTM is increasing, with isolates of different species now being researched in reference laboratories. Potentially pathogenic NTM that are present in the environment can be transmitted to humans or animals and can cause the onset of infection or disease [27]. Humans and animals are constantly exposed to NTM via inhalation or ingestion, resulting in the temporary or permanent colonization of the respiratory or digestive tract [28].

*M. gordonae* is extremely common in the environment and has been found predominantly in water. Although *M. gordonae* is considered to be a nonpathogenic bacterial species, infections have been reported in both immunocompromised and healthy patients [29]. Similarly, *M. flavescens* and *M. lentiflavum* have been frequently identified in clinical specimens, even without direct relation to disease. Nevertheless, studies refer *M. flavescens* in association with respiratory, skin and spread infections in humans, and *M. lentiflavum* causing fatal spread infections [30]. These data highlight the need for more research on these species [29,31].

The habitat of *M. mucogenicum* remains unclear. Evidence suggests that this species exhibits a ubiquitous distribution and that water is the main route of transmission. *M. mucogenicum* has been isolated from municipal water distribution systems, in which this microorganism can form biofilms and also infect amoebae and protozoa [28,32]. The high resistance of this species to chlorinated disinfectants and temperature extremes is notable, as these are major factors in the ecology of *M. mucogenicum*[32]. Although clinical isolates of this species are rare, these isolates have been associated with a wide spectrum of infections in both immunocompromised and healthy patients [30,32].

Since the last decade, the isolation of *M. vaccae* from environmental sources (such as soil and ponds) as well as animals (such as udder and skin lesions in cattle) has been reported [30]. *M. vaccae* is considered to be a nonpathogenic type of NTM and was recently ranked as a bacterium that is beneficial to the central nervous system. More specifically, in 2010, the American Society for Microbiology reported growth stimulation in certain neuronal types and consequently increased serotonin levels and decreased anxiety in mice exposed to *M. vaccae*[33]. Similarly, *M. parafortuitum* is not considered to be a pathogenic species of NTM. Phenotypically, *M. parafortuitum* resembles *M. fortuitum*, but certain biochemical differences between the two species motivated the classification of *M. fortuitum* as a novel species in 1966 [34].

*M. fortuitum* has been recovered from tap water, water distribution systems and various types of soil. In humans, *M. fortuitum* is associated with skin lesions but rarely causes pulmonary lesions or spread infections [30]. This mycobacterial species is the most common type of mastitis-associated NTM and was reported to be the etiological agent in 17 cases of mastitis in cattle [35]. The bacterium has also been isolated from raw-milk samples collected from bulk tanks [36], and in Brazil, *M. fortuitum* has been isolated from milk samples from cows with a positive reaction to the tuberculin test [37]. The occurrence of chronic and fibrosing mastitis, which is associated with *Mycobacterium* spp., is typically due to the excessive intramammary use of oily or antimicrobial drugs for mastitis treatment or is secondary to severe cases of clinical mastitis, as these bacteria act as opportunistic pathogens [35,38].

*M. smegmatis* also has been recovered from cattle with mastitis [39]. As the majority of the NTM, *M. smegmatis* is frequently found in water and soil [30]. Although this species may be associated with posttraumatic soft-tissue infections, *M. smegmatis* has not been described in disseminated infections, even in immunocompromised patients [30,40].

*M. immunogenum* is a newly described species belonging to the *M. chelonae*-*M. abscessus* group. This species mainly causes hypersensitivity pneumonitis secondary to aerosol inhalation. *M. immunogenum* is also associated with disseminated skin infections, septic arthritis, keratitis and nosocomial infections [41,42] and can form biofilms on environmental surfaces, such as pipes or equipment, resulting in a three- to 100-fold increase in disinfectant resistance [42].

*M. haemophilum* and *M. duvalii* are species associated with infection in immunocompromised patients or patients with human immunodeficiency virus (HIV) [30,43]. In contrast, *M. intracellulare* is an etiological agent of disease in healthy patients [30] that has been found in various treated and untreated water sources, demonstrating the bacterium’s ability to survive in water for extended periods [44]. This species typically causes pulmonary disease, followed by lung damage or reduced lung function, or cervical lymphadenitis, and occasionally affects children [30]. In contrast, *M. novocastrense*, first described in 1997, is rarely reported in humans, although there is evidence of this bacterium’s involvement in certain diseases [45].

*M. terrae* is often recovered from samples of soil, water and vegetables. Initially, this species was considered to be nonpathogenic, but several reports have associated this mycobacteria with cases of tenosynovitis and joint and pulmonary infections. Although *M. terrae* is rarely associated with disseminated infection, this species exhibits relatively high resistance to antimicrobial therapy [30,46].

The current study is the first report of the presence of *M. duvalii*, *M. haemophilum*, *M. immunogenum*, *M. lentiflavum*, *M. mucogenicum* and *M. novocastrense* in bovine raw-milk samples. The contamination of raw milk by mycobacteria is apparently inevitable, even under sanitary conditions, due to the ubiquitous nature of these microorganisms. Only the heat treatment of raw milk using commercial pasteurization protocols ensures the adequate destruction of mycobacterial contaminants. Thus, the transmission of viable mycobacteria to humans through heat-treated bovine milk is unlikely, whereas the consumption of raw bovine milk and related dairy products represents a public-health risk [38].

The increasing number of individuals worldwide who are infected with HIV in the world predisposes the increase in the number of cases of diseases that have been recognized as emerging and re-emerging, which are mainly those diseases caused by opportunistic agents, such as *Mycobacterium* spp. For example, in many regions of the world, tuberculosis is a major cause of death in HIV-infected individuals [47]. Such mycobacterial infections in HIV patients are frequently disseminated, although the transmission route of the bacteria remains controversial. As infection can occur in the lungs and/or gastrointestinal tract of HIV patients, a wide range of mycobacterial sources and modes of transmission must be investigated [48]. In particular, unpasteurized, mycobacteria-contaminated milk poses a serious risk to HIV patients.

The ecology and physiology of mycobacteria are complex and differ between MTBC and NTM. In recent years, the intersection of human, animal and mycobacterial ecology has exposed humans and animals to mycobacteria and has impacted mycobacterial ecology [48]. Research on the possible association between mycobacteria and the epidemiological factors preceding mycobacterial infection will be necessary to reduce the risk of exposure and infection.

## Conclusions

Microbiological cultures and PCR-based identification tests are possible tools for the investigation of the presence of *Mycobacterium* spp. in milk samples. Our data indicate that the Brazilian population may be exposed to mycobacteria through the consumption of raw bovine milk and related dairy products. These evidences reinforces the need to optimize quality programs of dairy products, to intensify the sanitary inspection of these products and the necessity of further studies on the presence of *Mycobacterium* spp. in milk and milk-based products.

## Abbreviations

HIV: Human immunodeficiency virus; M.: *Mycobacterium*; MTBC: *Mycobacterium tuberculosis* complex; PCR: Polimerase chain reaction; PNCEBT: ‘Programa Nacional de Controle e Erradicação da Brucelose e Tuberculose’; PRA: Restriction enzyme pattern analysis polymerase chain reaction; UNESP: Sao Paulo State University; NTM: Environmental nontuberculous mycobacteria; FAPESP: ‘Fundação de Apoio à Pesquisa do Estado de São Paulo’; CNPq: ‘Conselho Nacional de Desenvolvimento Científico e Tecnológico’.

## Competing interests

The authors declare that they have no competing interests.

## Authors’ contributions

MMJF: Conducted the mycobacterial culture-based procedures and the molecular identification of the species. Participated in the statistical analysis of the data. Drafted the manuscript. Read and approved the final manuscript. ACP: Conceived of and designed the study. Helped to draft the manuscript. Generally supervised the research group. Read and approved the final manuscript. MGR: Conceived of and designed the study. Helped to draft the manuscript. Generally supervised the research group. Read and approved the final manuscript. CQFL: Participated in the molecular identification of the species. Generally supervised the research group. Read and approved the final manuscript. JCFP: Performed the statistical analysis of the data. Read and approved the final manuscript. RGM: Acquired the samples and data on the population characteristics. Read and approved the final manuscript. ACBS: Participated in the molecular identification of the species. Read and approved the final manuscript. MM: Participated in the molecular identification of the species. Read and approved the final manuscript. FJPL: Participated in the mycobacterial culture-based procedures. Read and approved the final manuscript.

## Authors’ information

MMJF: M.V. M.Sc., School of Veterinary Medicine and Animal Science, Sao Paulo State University.

ACP: M.V. M.Sc. Ph.D., Professor at School of Veterinary Medicine and Animal Science, Sao Paulo State University.

MGR: M.V. M.Sc. Ph.D., Professor at School of Veterinary Medicine and Animal Science, Sao Paulo State University.

CQFL: Pharmaceutist, M.Sc. Ph.D., Professor at School of Pharmacy Sciences, Sao Paulo State University.

JCFP: M.V. M.Sc. Ph.D., Professor at School of Veterinary Medicine and Animal Science, Sao Paulo State University.

RGM: M.V. M.Sc., School of Veterinary Medicine and Animal Science, Sao Paulo State University.

ACBS: M.V. M.Sc. Ph.D., School of Pharmacy Sciences, Sao Paulo State University.

MM: Biologist M.Sc. Ph.D., School of Pharmacy Sciences, Sao Paulo State University.

FJPL: Laboratory Technician, School of Veterinary Medicine and Animal Science, Sao Paulo State University.
